# Digital Support for Patients Undergoing Bariatric Surgery: Narrative Review of the Roles and Challenges of Online Forums

**DOI:** 10.2196/17230

**Published:** 2020-07-15

**Authors:** Anna Robinson, Andrew K Husband, Robert D Slight, Sarah P Slight

**Affiliations:** 1 School of Pharmacy Institute of Population Health Sciences Newcastle University Newcastle Upon Tyne United Kingdom; 2 Institute of Population Health Sciences Newcastle University Newcastle Upon Tyne United Kingdom

**Keywords:** bariatric surgery, online forums, patient support, digital support, eHealth, mHealth

## Abstract

**Background:**

The internet has become an important medium within health care, giving patients the opportunity to search for information, guidance, and support to manage their health and well-being needs. Online forums and internet-based platforms appear to have changed the way many patients undergoing bariatric surgery view and engage with their health, before and after weight loss surgery. Given that significant health improvements result from sustained weight loss, ensuring patient adherence to recommended preoperative and postoperative guidance is critical for bariatric surgery success. In a patient cohort with high information needs preoperatively, and notoriously high attrition rates postoperatively, online forums may present an underutilized method of support.

**Objective:**

The aim of this study was to conduct a narrative review focusing on the developing roles that online forums can play for patients with bariatric conditions preoperatively and postoperatively.

**Methods:**

A literature search was conducted in October-November 2019 across 5 electronic databases: Scopus, EMBASE, PsycINFO, CINAHL, and MEDLINE. Qualitative or mixed methods studies were included if they evaluated patients undergoing bariatric surgery (or bariatric surgery health care professionals) engaging with, using, or analyzing online discussion forums or social media platforms. Using thematic analysis, themes were developed from coding patterns within the data to identify the roles and challenges of online forums for patients undergoing bariatric surgery.

**Results:**

A total of 8 studies were included in this review, with 5 themes emerging around (1) managing expectations of a *new life*; (2) decision making and signposting; (3) supporting information seeking; (4) facilitating connectedness: peer-to-peer social and emotional support; and (5) enabling accessibility and connectivity with health care professionals.

**Conclusions:**

Online forums could offer one solution to improving postoperative success by supporting and motivating patients. Future research should consider how best to design and moderate online forums for maximal effectiveness and the sharing of accurate information. The surgical multidisciplinary team may consider recommendations of online peer-support networks to complement care for patients throughout their surgical journey.

## Introduction

Digital technologies are recognized as an integral part of modern life. National Statistics estimate that 78% of adults own a smartphone, 90% of people regularly access home internet, and 20% of the population use wearable technologies such as smart watches and fitness trackers [1]. Not only are individuals readily using these technologies in their day-to-day lives [2], but also many are turning to them for support in managing their health and well-being. In the United States, 86% of the population are now connected online, with estimates reporting that 1 in 2 adults use the internet to seek information about their health [3].

One particular cohort that has benefitted from the advancing support of digital technologies is patients undergoing bariatric surgery. Obesity has been recognized as a global health concern, described as an *epidemic* by the World Health Organization (WHO). It is a chronic, life-limiting disease, which is associated with numerous serious health conditions including type 2 diabetes, cardiovascular disease, hypertension, sleep apnea, osteoarthritis, and some types of cancer (such as prostate, breast, ovarian, and pancreatic) [4,5]. The prevalence of bariatric surgery has increased alongside the rising trend in obesity across the Western world [4]. Bariatric surgery is often regarded as the most effective treatment for severely obese individuals [6], in whom evidence has suggested that weight loss can be up to 62% following the procedure [7]. However, it is well recognized that despite these promising outcomes, patients with bariatric conditions commonly experience challenges beyond the procedure itself in their bid for surgical success. Individuals may need to overcome social (eg, stigma), physical (eg, surgical complications), and psychological (eg, depression and negative body image) hurdles throughout their journey, in addition to adjusting to their new lifestyles (eg, recommendations for improved dietary intake and physical activity) following the procedure [8-10]. Furthermore, weight regain and inadequate weight loss have been recognized as obstacles impacting longer-term postsurgical outcomes [11]. This is where online forums have come into play, supporting patients throughout their surgical journey and beyond.

Online forums and telehealth platforms appear to have changed the way patients with bariatric conditions view and engage with their health before and after weight loss surgery [9,12]. The internet has become an important medium within health care, giving patients the opportunity to search for information, guidance, and seek social support. Previous studies have found links between social support and successful weight maintenance [13,14], improved quality of life, and increased patient empowerment [15-17].

We conducted a narrative review focusing on the developing roles that online forums can play for patients with bariatric conditions preoperatively and postoperatively. We also considered the broader challenges associated with online forums and the wider use of digital health technologies when it comes to supporting surgical patients.

## Methods

### Search Strategy

We conducted our search of the literature in October–November 2019 across 5 electronic databases: Scopus, EMBASE, PsycINFO, CINAHL, and MEDLINE. No limits were applied on publication dates. Bibliographies of all included studies were hand-searched and gray literature (using Google Scholar) identified additional papers. Keywords used in the searches covered the themes of bariatric surgery, online forums, and qualitative methodology. The full database search strategy and MeSH terms are available on request. All articles were exported to EndNote X9 (Clarivate Analytics) for data management.

### Inclusion Criteria

We included studies that had (1) included an investigation of patients undergoing bariatric surgery (or bariatric surgery health care professionals) engaging with, using, or analyzing online discussion forums or social media platforms, such as Facebook; (2) reported findings in the English language; and (3) conducted a qualitative or mixed methods study with qualitative transcripts of data available for analysis.

### Review and Thematic Analysis

**Figure 1:**
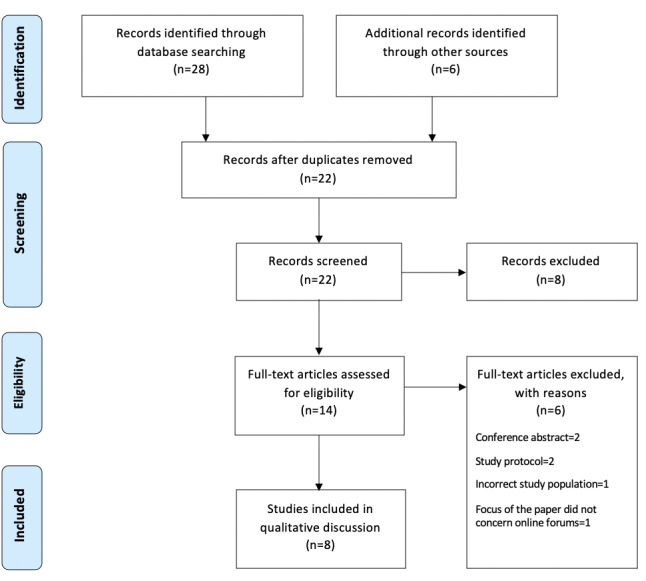
Flowchart of included studies.

Two authors (AR and AKH) reviewed the papers from the database search. Full texts were retrieved for articles that met the inclusion criteria or those that could not be rejected without certainty. The full texts were independently screened by AR and AKH. Any disagreements were resolved through discussion or by a third reviewer (SPS) where necessary. Figure 1 demonstrates the inclusion flowchart for this discussion.

Thematic analysis, as defined by Braun and Clarke [18], was performed by 2 researchers (AR and AKH) to identify patterns of themes in the data. Significant phrases and sections of available transcripts were coded with initial codes; these were then sorted and clustered into common coding patterns, which enabled the development of themes (derived from the data). Working iteratively and reflexively, the themes were reviewed and refined until they were coherent and distinctive [18]. Any discrepancies were resolved through discussion (AR and AKH) and, if agreement was not reached, by consensus with a third author (SPS). NVivo version 12 software (QSR International) was used for the organization of data and thematic analysis.

## Results

### Analysis of Search Data

The database searches returned a total of 28 papers. A further 6 records were included through gray literature and bibliography hand-searching. Following the removal of duplicates (n=12), 22 papers were screened and, of these, 8 were excluded based on their title and abstract. The remaining 14 full-text papers were assessed for eligibility, of which 6 were excluded with reasons. Eventually, 8 studies were included in this review. All 8 were published in the last 6 years and were conducted in the United States (n=4), Norway (n=2), Sweden (n=1), and Canada (n=1). Mixed methods were employed in 2 studies and the remaining used a form of qualitative methodologies, such as content analysis.

### Findings

Five themes relating to the roles of online forums in supporting patients undergoing bariatric surgery emerged: (1) managing expectations of a *new life*; (2) decision making and signposting; (3) supporting information seeking; (4) facilitating connectedness: peer-to-peer social and emotional support; and (5) enabling accessibility and connectivity with health care professionals.

#### Managing Expectations of a New Life

Life following bariatric surgery often requires a multitude of interpersonal adjustments, resulting in individuals creating expectations or goals for themselves to achieve following surgery. It is well-known within the literature that prior to surgery, patients with bariatric conditions may display unrealistic expectations of a *new life* following the procedure [19-21]. This appears to be a common finding among online forum preoperative postings, primarily with expectations focusing on the degree of weight loss individuals are hoping to achieve [22]. These patients have been known to perceive surgery as a *fix* or as a last chance for them to regain control over their weight when previous attempts by themselves in managing their weight have been unsuccessful [23]. This thinking may well link to poorly managed expectations from the side of the clinical team, but may also be a result of meeting certain eligibility criteria in order to qualify for the surgery [6]. Regardless of which, it was a common theme to see preoperative forum posts underpinned with emotions around excitement for an upcoming *new life* following surgery. Willmer and Salzmann-Erikson reported patients perceiving their surgery as a journey, whereby they change from their current weight and end with a happier, *lighter-weight life* [22]. These ambitious preoperative expectations appeared to go hand-in-hand with anticipation and nerves relating to undergoing the surgery itself. Willmer and Salzmann-Erikson reported how common it is for patients to anticipate dramatic changes of body and mind following weight loss surgery: “I look forward to the new me and my new life, I can barely wait”, and “Just think how unbelievably good it will feel afterwards” [22].

#### Decision Making and Signposting

This is a common theme in posts on preoperative forums related to surgical decision making; for instance, the suitability of surgery, the types of surgery on offer, and the impact of surgery on patient lifestyles [24]. Online forums enabled patients to seek relatable and supportive advice from other forum members. Atwood et al. [25] reported that responders reflected personally to these posts around decision making, using their own real-life examples to contextualize their choices: “I went with a bypass because I already had bad GERD [gastro-esophageal reflux disease], and the sleeve has been known to increase the amount of reflux you have”.

Deciding whether to undergo bariatric surgery is a big task for a patient to undertake, without considering the psychosocial impact that the surgery may have [26,27]. Online forums can play a role in supporting this decision making, where peers have come together to offer their thoughts and (often very personal) first-hand experiences of having gone through the surgery [25]. In their work, Ferry and Richards [24] acknowledged that patients felt similarities between themselves and other members’ stories, enabling them to put a real-life context behind their decision making: “I think my story is similar to many others I’ve read here ... I think I’m finally ready to seriously consider surgery, but I don’t know where to start”, and “I’m hoping to hear from all of you how surgery worked for you, so I can see if it can work for me”.

Preoperative patients were able to post and share information to help them weigh up the benefits and risks of going through the surgery; responders were seen to signpost their peers to alternative online sources of information to support their decision making “look at the National Institute of Health (NIH) website and journals such as New England Journal of Medicine” and “I looked at the percentage of probable weight loss. I thought this was a great tool for that: [website address]” [25]. Proactively seeking out digitally delivered information demonstrates the preoperative motivation of patients undergoing bariatric surgery and their acceptance of using online tools for support [28].

Preoperatively, patients also utilized online forums to seek advice and support about their choice of whether to *go public* with their surgery. The stigma of undergoing weight loss surgery is a common, and often underappreciated, hurdle that patients with bariatric conditions face [27,29]. With this in mind, it was not unusual to find posters reflecting on their personal decisions with other forum users: “I’ve chosen not to go public with this, except to family and certain friends. What have you done, have you told many people?” and “I’ve also chosen not to go public with what I’m about to do ... will do it little by little” [22]. It appears that emotional support closely links to surgical decision making, possibly affecting individuals more than is recognized within routine clinical practice. Having a way to openly and freely discuss this using online forums appears to be cathartic and beneficial for patients, with peers showing empathy and respect for those seeking preoperative support.

#### Supporting Information Seeking

Online forums can play a facilitative role in empowering patient engagement with their own care [30,31]. Having educational tools and support at their fingertips means that patients with bariatric conditions can actively seek out information at various stages of their surgical journey. For instance, this information may support patients to change their health behaviors prior to surgery, to learn about managing common symptoms following their surgery, or to normalize any ongoing emotions in postoperative life.

Preoperative and postoperative patients have been seen to readily post in online forums and lead discussion threads online [25,32,33]. Despite both sets of patients posting, there was a clear contrast between the nature of information being sought by preoperative and postoperative patients [22,32]. This mainly related to their own personal stage and accompanying information needs within the surgical journey. Preoperative patients used online forums for advice regarding physical preparation for their journey ahead, while also seeking information to normalize their emotions and nerves in the build-up to surgery [32]. Furthermore, it was common to see preoperative posts displaying a close affinity to the motivation and anticipation of a *new life* following surgery [22]. The patients were particularly keen to seek information about how they can improve the outcomes of their surgery. Preoperatively, patients were particularly receptive to advice given by postoperative patients who had recently gone through the surgical process.

These motivated information-seeking behaviors are demonstrated by patients postoperatively too; however, the content and type of information being sought differed. Unsurprisingly, following surgery many patients utilized the online forums to seek information to support their new diet and lifestyle. In a study by Das and Faxvaag [32], postoperative patients reported that they preferred to seek information via the online forum in comparison to liaising directly with their own medical team: “it’s easier to go on here [online forum] ask questions and get answers”. Their preferences may be related to the speed and ease with which answers can be obtained, given the high rate of engagement by forum users and their readiness to share information. In addition to this, postoperative patients have referred to more readily discussing sensitive issues on the forums as opposed to sharing these in a traditional face-to-face group or clinic appointment: “I think it is easier to talk about them [sensitive issues] in a place like this than face-to-face” and “you can be much tougher on the net, write things that you might not want to say to people because they are difficult to talk about. This becomes easier when you have a screen you can hide behind” [32].

#### Facilitating Connectedness: Peer-to-Peer Social and Emotional Support

It appeared that examples of peer support on online forums can take 2 forms, informational and emotional, with both types offered among preoperative and postoperative users [25,33]. Posts containing supportive advice aimed at those awaiting surgery appeared to feature heavily in American and Canadian preoperative forums [24,25,33-35]. They covered a range of content from advice on managing preoperative diet plans to tips relating to medicines following surgery, “you may want to pick up a pill crusher and a pill splitter in the drug store ... I had to crush and mix with drink in order to take [my medicines]”, and how to be best prepared for the emotional journey ahead of them, “keep your sense of humour. It’ll all be worth it in the end” [25]. Koball et al. [33] reflected in their mixed methods study, which analyzed content on a bariatric surgery Facebook page, that most preoperative patients used the forum to solicit answers to nutritional and medical questions (*P*<.001 for both). Postoperative patients were also seen to post on preoperative forums, offering their personal support as a *buddy* to someone who would be going on the journey: “I would be happy to make this journey with you” [25], “I would love to be your buddy” [25], “Believe me, I’ve been there ... feel free to message me with any questions” [24]. In their qualitative analysis of postoperative patients, Geraci et al. [34] reported the thoughts and perspectives of females who were 2 years postsurgery. Participants noted that their engagement with online support groups came from a want to inspire and give hope to the newbies (newly postoperative patients): “I want to give people hope that are just starting out and are thinking, ‘Will I ever lose the weight?’” [34]. It would be interesting to compare the prevalence of these posts on US forums with those from other countries, to assess possible cultural social norms.

#### Enabling Accessibility and Connectivity With Health Care Professionals

This is a smaller, yet significant, theme identified in the literature related to online forums connecting patients to health care professionals. In their study, Das et al. [36] evaluated the impact of an online forum on interactions between health care professionals and patients. They recognized the benefits in connecting the two groups to allow for easier access to evidence-based advice, as well as offering a convenient and geographically independent platform to promote patient engagement: “if we can get hold of them through this, then it’s really good. Because we want everyone to succeed”.

A lower threshold for information seeking by patients was also reported, with sensitive questions being more readily asked online as opposed to in face-to-face settings [36]. The forum also gave the health care professionals insight into the *day-to-day* lives of patients undergoing bariatric surgery, something that they would not normally see in a traditional, time-limited clinic appointment: “it’s obvious that one can capture things in the portal that I cannot capture during a consultation” and “you get more information about them here [online] than on the phone”.

## Discussion

### Preliminary Findings

This review has synthesized the findings from 8 studies focusing on the role and value of online forums for patients undergoing bariatric surgery. These early qualitative studies have shown how online forums can assist in supporting patients’ emotional and informational needs [37]. The value of peer-to-peer connectedness has been well documented in previous settings, with authors acknowledging benefits in quality of life and care satisfaction [38-40]. Not only does connectedness with peers allow for informational support, but it also provides emotional support and reassurance [41]. Online forums offer the opportunity to engage with a vast community of peers, which can be particularly beneficial for anyone who feels socially isolated [22].

Preoperatively and postoperatively, patients acknowledged the benefits and value of peer support in helping to maintain their own responsibility and motivation. This is not a new theme in the literature, where social connectedness and peer support have been linked to enhanced postoperative weight loss [14,42]. Atwood et al. [25] discussed that the frequency of informational peer support was higher in postoperative forums. They reported that posters readily shared their personal strategies as topics of information, such as ways to manage physical side effects or symptoms following surgery, and posting nutritional advice for adhering to lifestyle adjustments. The authors hypothesized that this information was likely to be reiterated from information provided at bariatric specialist appointments [25]. Given that a previous work has found that patients struggle with retaining information provided at specialist appointments [43], online forums could help to reinforce the ongoing educational messages throughout the surgical pathway.

It is well-evidenced that attendance at postoperative bariatric follow-up assessments is poor, with contributing factors relating to travel burden, geographical isolation, and time commitments [44-46]. Furthermore, patients have reported not seeing the value in postoperative clinics because the surgery had already been completed [47-50], and some preferred not to share sensitive information about their surgical journey in front of others [49]. Online forums can play a role in complementing traditional care and providing ongoing postoperative support, while helping to overcome these challenges. Studies have demonstrated that the content of online forums closely matched that of face-to-face clinics, meaning that patients are seeking support in the same subject areas [13]. Perhaps delivering this support via an online forum could be a way of overcoming these barriers, providing patients with the peer-support exposure they would be given if it were face-to-face, but ensuring anonymity for information sharing.

Internet-based forums, involving both health care professionals and patients, also existed in the wider literature, previously termed *online health communities* [31]. Patients have reported the benefits of utilizing these online forums for many health-related conditions, as well as for bariatric surgery [25,36,51,52]. In their review into the *empowerment effects* of online forums and support groups, Bartlett and Coulson [30] discussed the benefits of promoting active collaboration between the patient and their personal doctor [30]. The authors concluded that online forums increase patient empowerment and positively affect patient–provider encounters, leading to beneficial impacts on health-related outcomes and behavior change. Patients reported increased feelings of accountability and responsibility to adhere to healthier lifestyles and treatment plans as a result of digitally enabled connectivity with health care professionals [30]. These findings are also echoed in the wider health-related literature [37,53,54]. This receptivity toward positive health behaviors has also been associated with the concept of *teachable moments* [55,56]. A teachable moment is defined as “an event that creates an opportunity for positive behavioural change” [57]. Perhaps digital technologies and online forums hold value in this, where engagement with providers can opportunistically exploit patient insight to encourage healthy behaviors and empower to improved postoperative outcomes.

Despite the advantages of online forums, and digitally enabled health care, there are notable challenges too, particularly in understanding the digital divide and ensuring the accuracy of content and information being shared [51,58]. The digital divide refers to a gap in the access and use of technology [59,60], but with recent statistics supporting regular internet use by over 90% of the UK population, perhaps it could be better interpreted as “inequalities in understanding and interpreting the information” [1,61]. The digital divide has been acknowledged as a threat specifically to disadvantaged, minority, and older patients, as well as to those with lower sociodemographics and educational attainment [60,62,63]. In their review concerning the digital divide in health care, López et al. [62] call for the careful design and implementation of digital health interventions with the potential to eliminate disparities and bridge the digital divide, “we should ensure that disparities are not simply an afterthought for digitally enabled health care.” Despite increases in the integration of digital and online interventions, the digital divide is important to acknowledge in order to best support patients [61,64,65].

Sanders et al. [66] identified barriers to using online forums, reporting the main factors to be low health literacy, disinterest, and increased costs. Findings from a related study reiterate similar barriers as recognized challenges when it comes to the role of online forums for patients undergoing bariatric surgery [32]. We must not forget that there continues to be a population who prefer to use face-to-face contact with health care professionals or forms of traditional media (such as leaflets or books) as their primary source of health information [3,38,67]. Understanding the reasons behind this could be a pivotal finding in overcoming barriers to usability and uptake. This cohort should not be forgotten when it comes to introducing technology-delivered health care solutions; there is a risk of minorities falling further behind and widening the gap. Perhaps this supports the argument for implementing digital technologies (such as online forums) to complement traditional care, instead of replacing it.

Given the high acceptability (and engagement) of online forums, it would be prudent to consider the nature of the information shared, and the credibility and accuracy of the posts [31,33,35,68]. Many bariatric surgical forums are dominated by peer-to-peer communication without professional supervision or involvement. In their review, Li and Suh [69] reported that users associated credibility of posts with certain factors; increased presence of particular users (mainly how often they interact with posts) and posts that share anecdotes of personal experiences are perceived to be of higher credibility [69]. In another study, the content and accuracy of nutrition posts in a bariatric surgery Facebook group were evaluated [35]. The authors of that study raised concerns about the fidelity of the information posted, and encouraged health care professionals to caution patients when interpreting forum discussions [35]. They recognized benefits that may come from a greater presence of health care professionals in online groups, referring to potential roles in moderation of posts and provision of evidence-based recommendations [35]. Further to this, Lindsay et al. [70] reported that having a moderator in an online support group for heart disease meant patients were more likely to adhere to advice, and thus more readily maintaining healthy behaviors. Similar findings were reported by Graham et al. [9], but this time from the perspective of a bariatric surgical health care professional [9]. Members of the surgical team specifically acknowledged that information shared which originates from other countries may conflict with the advice from UK recommendations, and that discussions about dietary intake may not be adequately tailored for those recovering from bariatric surgery [9].

It is clear that online forum content is an area that would benefit from further research in order to systematically review the data and better appreciate the place of digital support in a modern health care system. Surgical team members should consider the availability of digital support, and the possibilities or detriments this could have on patients before and after surgery.

### Conclusion

In a patient cohort with notoriously high attrition rates at postoperative follow-up, and vastly changing needs during their surgical journey, the potential of online forums may well be an untapped method of support. Online forums could offer one solution to improving postoperative success by supporting and motivating patients. Future research should further explore the value of online forums and their place within modern health care systems. Involving patients to determine the optimal design and moderation of online forums will help to maximize usefulness and effectiveness. Members of the bariatric surgery multidisciplinary team may consider recommendations of peer-support networks to complement care for patients throughout their surgical journey.
